# Prime Vaccination with Chitosan-Coated Phipps BCG and Boosting with CFP-PLGA against Tuberculosis in a Goat Model

**DOI:** 10.3390/ani11041046

**Published:** 2021-04-08

**Authors:** Yesenia Guadalupe Contreras-Magallanes, Marina Durán-Aguilar, Susana L. Sosa-Gallegos, Ángel H. Álvarez, Fátima A. Andrade-Santillán, Isabel Bárcenas-Reyes, Sara González-Ruíz, Elba Rodríguez-Hernández, Germinal J. Cantó-Alarcón, Feliciano Milián-Suazo

**Affiliations:** 1 Doctorado en Ciencias Biológicas, Universidad Autónoma de Querétaro, Avenida de las Ciencias S/N, Juriquilla, Delegación Santa Rosa Jáuregui, 76230 Querétaro, Mexico; yesconmag@hotmail.com; 2 Facultad de Ciencias Naturales, Universidad Autónoma de Querétaro, Avenida de las Ciencias S/N, Juriquilla, Delegación Santa Rosa Jáuregui, 76239 Querétaro, Mexico; marina.duran@uaq.mx (M.D.-A.); susana.lucia.sosa@uaq.mx (S.L.S.-G.); isabel.barcenas@uaq.mx (I.B.-R.); sara.gonzalez@uaq.mx (S.G.-R.); gcanto07@uaq.mx (G.J.C.-A.); 3 Centro de Investigación y Asistencia en Tecnología y Diseño del Estado de Jalisco, Av. Normalistas 800, Col. Colinas de la Normal, 44370 Guadalajara, Mexico; aalvarez@ciatej.mx; 4 Maestría en Salud y Producción Animal Sustentable, Universidad Autónoma de Querétaro, Avenida de las Ciencias S/N Juriquilla, Delegación Santa Rosa Jáuregui, 78230 Querétaro, Mexico; a.andrades7@yahoo.com; 5 Centro Nacional de Investigación Disciplinaria en Fisiología y Mejoramiento Animal, INIFAP, SAGARPA, Km. 1 Carretera a Colón, Ajuchitlan, 76280 Colón, Mexico; rohe577@hotmail.com

**Keywords:** *Mycobacterium bovis*, BCG vaccine, chitosan, poly (lactic-co-glycolic acid), cattle, goats

## Abstract

**Simple Summary:**

Bovine tuberculosis is a disease that affects cattle and other animal species worldwide and represents a risk to public health. Even though there is a vaccine that has been used to control tuberculosis in humans for almost 100 years, up to now, it has not been used in animals. The reason is that vaccination interferes with the tuberculin test, the current test to diagnose tuberculosis in the field, and shows an inconsistent efficacy in animals. Recent studies report that prime vaccinating with BCG and boosting with proteins vaccinations perform better. In addition, there are reports that some polymers increase the immune response against various infectious diseases; therefore, testing a vaccine formula with polymers sounds like a wise thing to do. In this study, we showed that priming with BCG and boosting with a culture filtrate protein, alone or in combination with a polymer, the number of animals with lesions, the number of lesions per animal, and the size of the lesions in vaccinated animals, compared with those not vaccinated or those vaccinated with BCG alone, are significantly reduced. Our results mean that a vaccination used as a complement of actual tuberculosis control programs in animal populations can be useful to reduce tuberculosis dissemination.

**Abstract:**

Attempts to improve the immune response and efficacy of vaccines against tuberculosis in cattle, goats, and other animal species have been the focus of research in this field during the last two decades. Improving the vaccine efficacy is essential prior to running long-lasting and expensive field trials. Studies have shown that vaccine protocols utilizing boosting with proteins improve the vaccine efficacy. The use of polymers such as chitosan and PolyLactic-co-Glycolic Acid (PLGA) improves the immune response against different diseases by improving the interaction of antigens with the cellular immune system and modulating the host immune response. This study shows that the prime BCG vaccination, boosted with a culture filtrate protein (CFP), alone or in combination with chitosan and PolyLactic-co-Glycolic Acid (PLGA), have the potential to reduce tuberculosis (TB) dissemination by reducing the number of animals with lesions, the number of lesions per animal, and the size of the lesions in vaccinated animals, compared with those not vaccinated or those vaccinated with BCG alone. The vaccinated groups showed significantly higher Interferon-γ levels in the blood compared to the control, nonvaccinated group after vaccination, after boosting, and after the challenge with the wild-type *Mycobacterium bovis* strain.

## 1. Introduction

Bovine Tuberculosis (bTB) remains a serious problem for livestock worldwide, especially in developing countries, where the strategy of “test-and-slaughter” is not economically viable [1,2,3,4]. The prevalence of bTB in dairy cattle in not developed countries is especially high; therefore, the testing and disposing of cattle would represent high costs to the dairy industry, rendering this practice unfeasible. Lately, one of the alternatives recommended for reducing the prevalence of this disease in animals has been the use of the vaccine BCG, alone or in combination with specific proteins [5,6,7]. BCG has been used in humans for nearly a century; however, it has not been used in cattle to date [8,9]. For the last 20 years, the vaccination has been evaluated experimentally in different animal species. It has been tested in cattle [8,10,11,12,13,14,15,16] and, also, in whitetail deer [17], badgers [18], brushtail possums [19,20], and goats [21,22,23,24,25,26].

Experimental studies to evaluate the vaccine’s efficacy against TB in cattle are expensive and require complex premises to maintain and handle the experimental animals. Testing in goats is less expensive and has been considered as an alternative model for testing prototype vaccines against TB in humans and animals [27,28]. It has been shown that goats develop TB lesions similar to those observed in cattle and humans [28,29]. Since goats are also ruminants, the immune response is expected to be similar to that observed in cattle. Implementing vaccine strategies in current TB control programs in countries where “test-and-slaughter” is not feasible is the wheel that moves research to improve the efficacy of BCG, the only authorized vaccine currently used in humans.

Studies in cattle have shown that the vaccination reduces transmission by decreasing the number of animals with lesions, the number of lesions per animal, the size of the lesions, and tissue bacillary loads [7,8,30]. It has been reported that protection is better when calves are vaccinated early after birth compared to at two–four weeks of age with 10^5^–10^6^ (Colony-Forming Units) (CFU), with a possible revaccination between one and two years to maintain the appropriate levels of immunity. No differences in the efficacy have been observed between the Danish and the Pasteur strains, the most frequently used vaccines in the world. The vaccine is safe, but while it does not exacerbate the infection, it also does not cure it [8,10,11,12,13,14,15], and the vaccination of pregnant cows does not represent a risk for abortion [16]. Boosting with culture filtrate protein (CFP) increases the immune response and protection against pathological damage [8,16,31].

Therefore, prime BCG vaccination with a CFP boost is currently being considered in many countries as a complement to existing control programs to reduce the incidence of bTB [32]. Finding the means to increase the vaccine efficacy by improving and sustaining, the protective immune response for longer periods [33] has been a goal of many research protocols. The idea is to increase the interaction of the antigen with the immune cells, for example, by encapsulating and releasing antigens to modulate the host immune response [34]. There is sufficient evidence that the combination of adjuvants with BCG enhances the BCG immunogenicity and protection against TB. Different elements such as Rapamycin, Lactoferrin, and the agonists of Toll-Like Receptor (TLR) 7 and 9 increase the immune response to BCG, activating CD4+ and CD8+ T cells to increase protection in mice [35,36]. Some reports have shown that nanocoating BCG with polyinosinic-polycytidylic acid (poly I:C) and chitosan increase the cell-mediated immunity with no effect in the vaccine viability in vitro [33]. Polymers have low toxicity, are easy to obtain, and are highly biodegradable. They are also good antibacterial agents, with a positive effect on the treatment of TB [37,38]. Biodegradable polymers such as chitosan and PolyLactic-co-Glycolic Acid (PLGA) have been used as vaccine adjuvants to stimulate the immune system and as carriers for vaccine delivery [39].

Chitosan is a cationic polysaccharide comprising copolymers of glucosamine and N-acetylglucosamine obtained from exoskeletons of crustaceans, yeast, and fungi [40]. It is insoluble in alkaline and neutral pH but forms salts with inorganic and organic acids and is available in a range of molecular weights. Chitosan salts bind strongly to negatively charged materials such as cell surfaces and mucus. It is bio-adhesive and significantly increases the half-life clearance of antigens [41], where adjuvants enhance the uptake of antigens by macrophages [42] and induce the production of cytokines such as interleukin and interferon. There is evidence that TB vaccines with chitosan induce strong protective and cell-mediated CD4+ and CD8+ immune responses in animal models and, when employed as a booster, enhance the protection against TB infection in mice [42,43,44,45]. Chitosan solutions create an antigen depot, and more than 60% of a protein antigen delivered in chitosan remains at the injection site for seven days [45].

PLGA is a synthetic polyester that degrades into lactic and glycolic acids. PLGA stimulates both humoral and cellular immune responses [46,47]. The use of antigens and adjuvants in a formulation of slow-release particles increases the vaccine efficacy by enhancing the availability of antigen-to-antigen-presenting cells [48]. It is a potent inducer of TH1, which is associated with protection against TB infection, and TH17 responses [48,49,50,51]. PLGA-covered antigens quickly escape from the endolysosomes and are transported to the cytoplasm, preventing the lysosomal degradation of null fragments, enhancing, in this manner, the protective efficacy of the vaccine [44,52].

Vaccine experiments in cattle have demonstrated that low doses of BCG, 10^3^–10^6^ CFU, induce a greater protection than higher doses [53], that pre-exposure to environmental *Mycobacterium* can negatively affect the vaccine efficacy [54], and that the vaccination of neonatal calves induce higher levels of immunity than those observed in calves vaccinated at five to six months of age [6,8,13,55]. As in cattle, trials have revealed that goats vaccinated with BCG or other experimental TB vaccines have a significant higher interferon-gamma release, fewer lesions, and lower bacterial culture loads than those unvaccinated [25,26,56,57]. Therefore, the purpose of this study was to evaluate different BCG vaccine formulations using the BCG prime vaccination, alone or chitosan-coated, and CFP boosting, alone or combined with chitosan and PLGA, in a goat model.

## 2. Materials and Methods

Experimental animals. Thirty-five, 3 to 5 months of age Alpine-Nubia breed goats deriving from a TB-free area were included in the study. The animals were randomly assigned to five experimental groups of seven animals each. Animals were then placed in experimental units especially designed for this purpose, with enough space and shade and food and water at libitum. Two animals died 1 week after the experiment started due to diarrhea caused by a coccidian. At that time, all animals were treated with Baycox^®^ (Bayer) at a dose of 20 mg/kg. One animal from the control group died 1 week prior to slaughter; in a necropsy, numerous lesions compatible with TB were observed in the lungs. All animals tested negative for paratuberculosis in an ID screen^®^ diagnostic kit for the serum and plasma samples (LABGENE Scientific SA, Châtel-Saint-Denis, Switzerland). An experienced goat caretaker oversaw the monitoring and feeding of the animals on a daily basis. Experienced veterinarians from our working group performed the handling, tuberculin testing, and sampling of the experimental animals. All animals were confirmed negative to TB by the tuberculin caudal-fold and by the Interferon-γ (IFN-γ) assay (Bovigam, Prionics AG, Zurich, Switzerland) tests.

BCG vaccine strain. The Phipps strain (American Type Culture Collection (ATCC^®^) 35744™) was selected based on the results from a previous study in which it was the most efficient in providing protection against pathologic damage after a challenge in mice where 10 BCG daughter strains were compared [58]. The strain was purchased from the American Type Culture Collection (ATCC, Manassas, VA, USA). Briefly, reactivation of the BCG was performed as follows: 300 mL of culture medium containing 1.41 g of Middlebrook 7H9 base medium (BD, Franklin Lakes, NJ, USA) and 1.32 g of sodium pyruvate (Golden Bell, crystals 110.05) in 270 mL of distilled water with 0.6 mL of glycerol. This was autoclaved at 121 °C for 10 min. After sterilization, it could cool to 50–55 °C, and then, 30 mL of ADC (BD BBL Enrichment for Middlebrook) was added in a laminar flow hood. This medium was incubated at 37 °C for 48 h to verify sterility. After that, the original bacterial pellet containing the *Mycobaterium bovis* BCG Phipps strain was added, and this incubated at 37 °C for 6 weeks. Subsequently, this was passed to a Stonebrink and Lowenstein Jensen medium and incubated at 37 °C until growth occurred at 6 weeks.

BCG Phipps *M. bovis* growth. The vaccine strain was grown in enriched culture Middlebrook medium 7H9 broth + ADC + 20% Tween 80. A total of 1000 mL of culture medium was prepared with 4.7 g of Middlebrook 7H9 base medium (Difco™) and 900 mL of distilled water with 2.5 mL of 20% Tween 80 (Sigma-Aldrich, St. Louis, MO, USA). This was then placed in a flask and autoclaved at 121 °C for 10 min. Once sterilized, it was allowed to cool to 50–55 °C, and then, 100 mL of ADC was added with a 0.2-μm Nalgene syringe filter in a laminar flow hood. The medium was incubated at 37 °C for 48 h to check for sterility. Then, several *M. bovis* colonies from the Stonebrink and Lowenstein‒Jensen reactivation culture media were taken, passed into these media, and incubated at 37 °C for 4 weeks. Then, 1000 mL of culture medium was prepared to contain 4.7 g of the Middlebrook 7H9 based medium (Difco™) in 900 mL of distilled water with 2 mL of glycerol and was placed into four 250-mL flasks and autoclaved at 121 °C for 10 min. Once sterilized, this could cool to 50–55 °C, and then, 100 mL of ADC sterilized by a 0.2-μm Nalgene syringe filter in a laminar flow hood was added. This medium was incubated at 37 °C for 48 h to verify the sterility. At about 4 weeks, the colony growth was observed. These cultures were then centrifuged (20 min × 1008 *g*) and added to a (SPGA) solution at a 1:1 concentration with sterile PBS for bacterial separation with a 23-G needle. Bacterial count and dose preparations were performed and maintained at −70 °C until their use.

BCG chitosan coating. The chitosan suspension was prepared at a 0.001% concentration, where 500 mg of low molecular weight chitosan (Sigma-Aldrich, St. Louis, MO, USA) and 4.5 g of sodium chloride (NaCl) were added to 500 mL of water with 5 mL of acetic acid at pH 6. A BCG Middlebrook 7H9 culture medium was used for BCG chitosan coating. It was first centrifuged (10 min × 1008 *g*); then, the pellet was washed twice with 0.9% NaCl and sonicated in a water bath for 10 min to separate the clumped bacteria. The suspension obtained was passed 10 times through a 23-G needle to break up the bacterial clumps. During this step, the chitosan solution (0.5 mL/mL) was added and was maintained in an orbital shaker for 20 min. The new solution was washed twice with 0.9% NaCl and passed once more through a 23-G needle to separate the coated bacteria. This bacterial suspension was added to the Middlebrook 7H9 + ADC + glycerol culture medium and left in incubation for 4 weeks. To verify that chitosan is harmless for BCG bacilli in vitro, a bacterial viability test was performed by growing BCG and BCG chitosan coated in a Middlebrook 7H9 + ADC + glycerol medium. On day 31, 100-µl samples of the culture medium were obtained for electron microscopy scanning. BCG and BCG chitosan-coated suspensions were isolated in a Stonebrink solid medium by serial dilutions ranging from 10^1^–10^8^ to obtain the CFU/mL. The cultures were incubated at 37 °C for 4 weeks with checks each week to verify the culture growth.

CFP production and CFP-PLGA coating. The field strain obtained from a cow’s lymph node lesion was grown in Stonebrink solid medium. After 8 weeks, the colonies were harvested and placed on the wall of a flask wall of a Middlebrook 7H9 + ADC + glycerol medium. This culture was centrifuged for 10 min at 1008 *g*. The supernatant passed three times through a 0.2-μm Nalgene syringe filter and then transferred through a molecular weight filter (Millipore 4307 Centriprep YM-30, 30-kDa NMWL) to obtain a final volume of 60 mL. Protein quantification was performed with the Biuret reagent (MEYER TG1118618 Reagent) for a final CFP protein concentration of 1250 μg/μL.

Encapsulation of the CFP was performed with 1 mL of CFP in 3.7 mL of a 3% solution of Poly (D, L-lactide-co-glycolide) lactide:glycolide; PLGA) (75:25; Sigma-Aldrich, St. Louis, MO, USA) and chloroform, which was then stirred in a vortex for 1 min to form the first emulsion. This emulsion was added with Poly (Vinyl Alcohol) (PVA; Sigma-Aldrich, St. Louis, MO, UA; 87–89% hydrolyzed) at 10% at a 1:1 ratio and maintained under magnetic stirring for 24 h. This was then stirred in a PowerLyzer (2800G with two cycles of 0.45 s and a rest of 0.30 s) and centrifuged for 10 min at 11,200 *g*. The supernatant was removed to quantify the nonencapsulated protein with a Biuret reagent. The pellet was washed three times with distilled water for 5 min at 11,200 *g*. Finally, it was suspended in sterile PBS. A sample of 100 µL was taken for electron microscopy scanning. The CFP booster formulations were prepared with 900 μL of CFP and 100 μL of adjuvant (Montanide^TM^) and oil-based adjuvant composed of a natural metabolizable oil and a highly refined emulsifier from the manide monooleate family [59].

Vaccination, boosting, and challenge: Animals were allocated throughout a completely randomized experimental design into five experimental groups (Table 1). Vaccinated groups were inoculated subcutaneously on the right side of the neck with a dose of 1 × 10^3^ CFU and the chitosan-coated BCG at 1 × 10^2^ cells in a 2-mL diluent at week one. Boosted groups were inoculated subcutaneously 4 weeks after prime BCG vaccination with 720 μg/μL of CFP inoculated with the different boosting protocols. The vaccine dose was determined as lower to those reported in previous studies in goats to prevent premature deaths [25,26,56]. The adjuvant used was Montanide^TM^ as 10% of the used formula. The challenge inoculum was prepared with a mid-log phase of a wild-type *Mycobacterium bovis* grown in Stonebrink + pyruvate culture medium. The challenge strain was collected from a cow’s lymph nodes in 2010 in Mexico and was maintained in glycerol at −70 °C. Bacilli were pelleted by centrifugation at 750 *g* and washed twice with a Phosphate-Buffered Saline solution (PBS; 0.01 M, pH 7.2). This was then homogenized in PBS and shaken with glass beads continuously at 200 rpm for 1 h. The homogenate was sterile-filtered twice through a 40-µm syringe filter and diluted to the established doses (1 × 10^6^ CFU) in 0.5 mL of PBS. Goat kids sedated with 0.25 mg/kg xylaxine were challenged by direct inoculation into the trachea using a 3-mL (23G × 25 mm) syringe.

**Figure 1:**
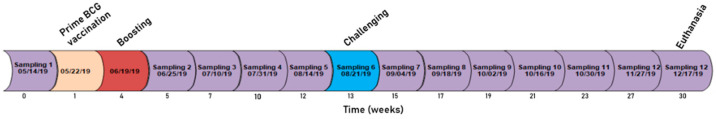
Dates of sampling, BCG prime vaccination, boosting, challenging, and euthanasia of vaccinated and nonvaccinated animals against tuberculosis in a goat model.

Blood sampling and antigen stimulation. Blood samples for the IFN-γ release assay were collected every 2 to 3 weeks until the end of the experiment (Figure 1). Blood samples (*n* = 13) were collected from the jugular vein and placed in heparin tubes. Then, 750 µL of the whole blood of each animal was incubated on microplates in duplicate with 50 mL of each antigen; that is, bovine-purified protein derivative (PPD) and avian PPD. PBS was used as negative control for each animal tested and 50 mL of pokeweed mitogen of a 1-mg/mL concentration solution (Sigma-Aldrich, Gillingham, UK) as the positive control. Microplates were then incubated in a humidified 5% CO_2_ incubator at 37 °C for 20 h. Optical Densities (OD) of PBS from the control wells were employed to normalize individual readouts and to calculate the OD. The final OD readings were obtained by subtracting sample readings from the PBS control readings. The IFN-γ release in whole-blood cultures after 16 h in vitro were performed on a commercial bovine IFN-γ microplate Enzyme-Linked ImmunoSorbent Assay kit (ELISA; Bovigam^®^; Prionics AG, USA).

**Figure 2:**
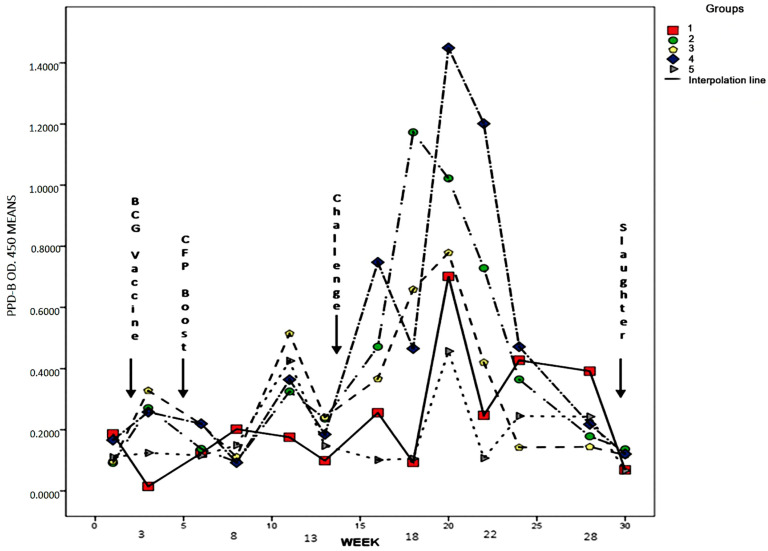
Antigen-specific Interferon gamma (IFN-γ) response in goats after vaccination, boosting, and challenging with a *Mycobacterium bovis* wild-type strain. The purified protein derivative (PPD) bovis was used in the stimulation of whole blood in vitro. IFN-γ release values per group are expressed as the mean Optical Densities (OD 450 nm).

Slaughter and lesion scoring. About 6 months after the challenge, experimental animals were sent to slaughter for carcass inspection and lesion scoring and for tissue sampling collection for histopathological and microbiological analyses. The animals were euthanized by electro insensitization following NORMA Oficial Mexicana NOM-033-ZOO-1995 Guidelines, Sacrificio humanitario de los animales domésticos y silvestres (Mexican Official Norm for the Humanitarian Slaughter of Domestic and Wildlife Animals). Criteria for animal scoring are presented in Table 2. Instead of counting or classifying individual lesions, the score for the whole animal was based on the magnitude of tissue damage in affected organs and the characteristics of the lesions. To determine the presence and magnitude of the lesions, all organs were carefully removed and sliced. Complete lymph nodes were removed; one-half of these was frozen for bacteriologic analysis, and the remaining half was placed in a 10% formalin buffer solution for histopathological analysis. In addition, about four square centimeters of the cranial lung’s lobe from all animals were collected for bacteriological and histopathological analyses. In order to perform a careful inspection of the carcasses, the animals were slaughtered over a 2-week period, one animal randomly selected from each experimental group at the same time. Carcass inspection focused on the lymph nodes of the head (retropharyngeal), thorax (mediastinal and tracheobronchial), abdomen (mesenteric), lungs, and liver; examples of score values for the lesions are found in Figure 2. At slaughter, the number and the treatment group of the animals were blinded to the veterinarian who scored the lesions.

Statistical analysis. The average IFN-γ concentration (OD raw data) per experimental group for each sampling period was compared with a one-way Analysis of Variance (ANOVA) test. The average lesion scores per group were compared with the H statistic in the Kruskal‒Wallis test. A *p*-value of ≤0.05 was considered significant. A relation between the number of lesions and the animal lesion scores was determined with Spearman’s correlation coefficient. All statistical analyses were performed with SPSS version 22 statistical software.

## 3. Results

With the exception of some sneezing associated with powder derived from the food provided (a combination of ground forage, alfalfa, and corn), no clinical sign suggestive of TB was observed in the experimental animals. All animals, including those with high scores of lesions at postmortem, had a good body condition and life behavior. At slaughter, only one animal exhibited a subcutaneous TB lesion at the vaccine injection site, suggesting that the inoculation method worked well.

### 3.1. IFN-γ

The mean IFN-γ release, and the ANOVA *p*-value for the comparison of groups at each sampling week, measured as 450-nm optical density (OD) in an ELISA test, are depicted in Table 3. There was no significant difference between the experimental groups at sampling weeks one, three, and six (*p* > 0.05), even though group 1 had a lower IFN-γ release than the vaccinated groups, groups 2–5. Nor was there any significant difference between the groups at the end of the experiment at weeks 24, 28, and 30 (*p* > 0.05), when the IFN-γ release returned to the baseline levels. In general, there was a significant difference (*p* < 0.05) between the vaccinated and the control groups at sampling weeks 8–22, except for certain weeks during which the difference was variable, and there was no significant difference between some of the vaccinated groups and the control (Table 3). The peak IFN-γ release was reached at week 20 in all of the experimental groups. Five weeks after the challenge, groups 5, 4, 2, and 3, in descending order, had the best IFN-γ responses, and the controls exhibited lower IFN-γ responses. All groups returned to the baseline levels at week 30 (Figure 2).

### 3.2. Lesions in the Animals

The number and the proportion of animals with lesions and the average score of the lesions per experimental group are listed in Table 4. All animals in group 1, and six of the seven animals in group 2, had lesions at slaughter. Only two of seven animals from groups 3 and 4 had lesions. Group 5 had four out of six animals with lesions. A significant difference (*p* < 0.05) was observed between groups 3 and 4 compared with groups 1 and 2. This result shows that groups 3 and 4 perform better than the rest of the groups in protecting against the development of lesions of TB.

**Figure 3:**
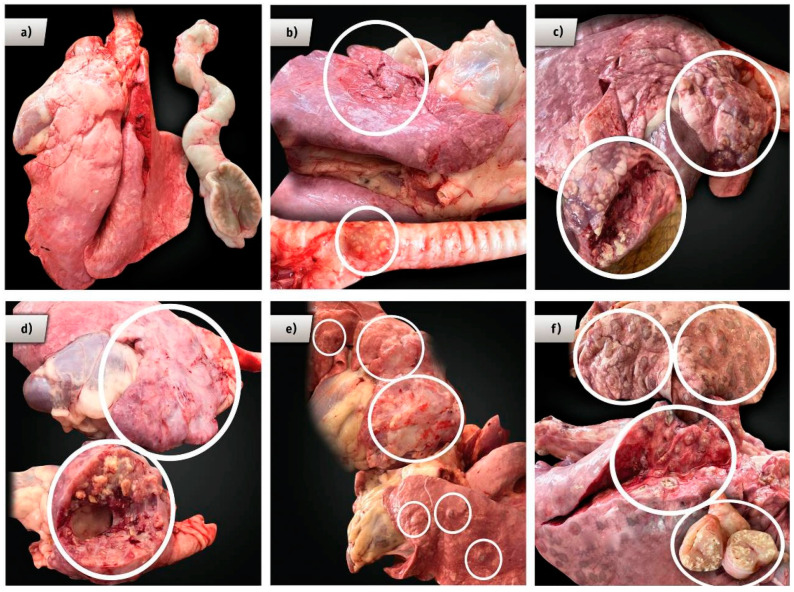
Illustration of the score values in experimental animals vaccinated against tuberculosis (TB) with different vaccine protocols and challenged with a *Mycobacterium bovis* wild-type strain. (**a**) Score 0, no visible lesions, (**b**) Score 2, small focal purulent tuberculous lesion in the lung’s cranial lobe, (**c**) Score 4, medium-sized purulent tuberculous lesion in the lung’s cranial lobe, (**d**) Score 6, large purulent tuberculous lesion in pulmonary cranial lobe, (**e**) Score 8, multiple small focal tuberculous lesions in lungs, and a medium-size purulent lesion in the cranial lobe, and (**f**) Score 10, multiple small tuberculous lesions covering the lungs and large lesions in the pulmonary cranial lobe and the lymph nodes.

The mean lesion scores for all experimental groups are also to be found in Table 4, and examples of the specific lesion scores are illustrated in Figure 3. No significant differences were observed for the lesions’ average scores between group 1 (6.3 ± 3.2) and the BCG-alone vaccinated group (3.0 ± 2.2) in a post-hoc Tukey’s test. In addition, no significant difference was observed either among all of the vaccinated groups, 2–5 (*p* > 0.05). Average lesion scores for groups 3–5 (1.1 ± 2.2, 1.4 ± 3.2, and 1.7 ± 1.5, respectively) were considerably lower than that in group 2, the BCG-alone vaccinated group. This result shows that the polymers chitosan and PLGA in the vaccine formula made no significant differences compared to that shown by the BCG prime-vaccinated and CFP-boosted formula (group 3).

**Figure 4:**
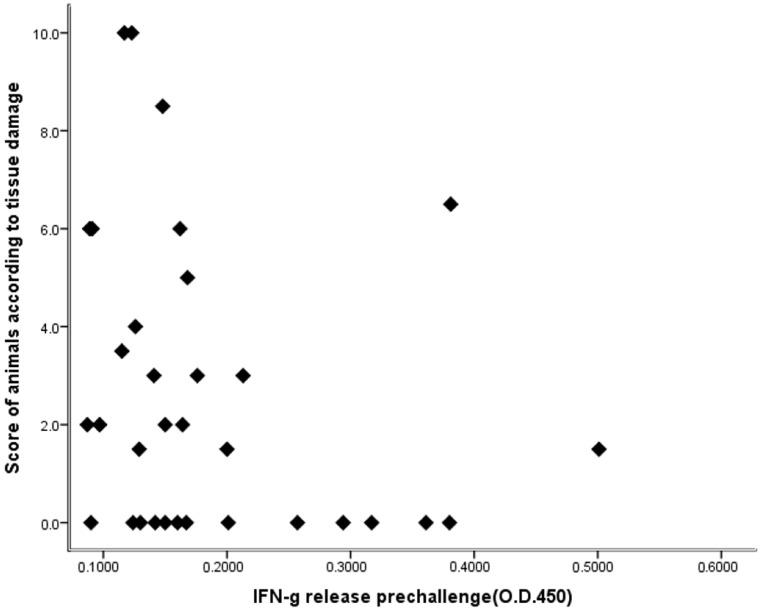
Relationship between the blood IFN-γ release 1 week prior to challenge and the lesion scores of the experimental animals vaccinated against TB with a BCG strain and challenged with a *Mycobacterium bovis* wild-type strain.

One important issue in TB vaccine testing in animals lies in the identification of surrogates for vaccine efficacy. To date, the best option is the determination of IFN-γ in the blood [16,60]; the concentration of IFN-γ is associated with the development of lesions in infected animals. In this study, no significant relationship (r = −0.250; *p* = 0.16) was found between the blood‒IFN-γ concentration in the week prior to the challenge and the presence of a lesion at slaughter (Figure 4).

## 4. Discussion

The experimental efficacy of BCG to reduce the pathological damage in vaccinated animals against tuberculosis in cattle and other animal species has shown some variations [10,11,19,53,61,62]: factors such as the vaccine strain, doses, and route of inoculation [63] play a role. In this study, we used the Phipps strain, because it showed better protection than other BCG strains in a mice model [58]. However, most studies have used the Pasteur or the Danish strains and found no differences in the protection [12,14,15,64]. Concerning the vaccine dose (1 × 10^3^ CFU for BCG alone and 1 × 10^2^ CFU BCG chitosan-coated), which was somewhat lower than that utilized in other studies [22,23,25], was to prevent the sudden death of the animals and have the opportunity to evaluate how long the immunity lasted in the vaccinated animals. Based on the high level of pathological damage observed in the control group, this dose is adequate for experimental studies in goats and for preventing the development of tuberculous lesions in vaccinated animals.

Different routes of inoculation have been used to test the vaccine efficacy [17,55]. In this experiment, we utilized the subcutaneous (sc) route, because, as others proposed, we believe that would be the most practical route if a massive vaccination of cattle were to be approved [11,61]. Previous studies have reported the role of boosting in the immune response to vaccination [10,11,65,66,67,68,69]. Our results agree with those reports: all of the boosted groups demonstrated a better performance in lesion scores than the BCG-alone vaccinated group. It is interesting that group 3, which was primed with BCG and boosted with CFP plus an adjuvant with no polymers added, exhibited the best performance in preventing pathological damage, suggesting that this protocol is sufficient to reduce disease and, in consequence, TB dissemination. This result agrees with the previous findings reported by our group in experiments with cattle [8].

A significant difference in the lesion scores’ average was observed between group 1; the control group; and the vaccinated boosted groups (groups 3, 4 and 5; Table 4). This difference could have been greater; one animal in the control group showed a large tuberculous lesion at the challenge site, suggesting that the inoculum were injected out of the trachea, possibly reducing this difference. Thus, the animal showed few lesions in the lungs and lymph nodes at slaughter. None of the remaining animals demonstrated a lesion as large as this at the challenge application site. IFN-γ production has been employed as a surrogate for vaccine efficacy [10,70,71,72]; however, there is a trend that animals with high IFN-γ release have less lesions [73,74,75]. In this study, we found that the correlation between IFN-γ release and the lesion score was not significant (r = −0.250; *p* = 0.16, Figure 4); contrary to the findings by our group working with cattle [8], where this relationship was significant (*p* < 0.05).

No statistical difference in the lesion scores’ average was observed among groups 2–5, the vaccinated groups (Table 4). However, group 2, that vaccinated with BCG alone, was not statistically different from group 1, while in groups 3–5, the boosted groups were statistically different. This result agrees with previous reports in which it was shown that boosting significantly increases the protection against the pathological damage of TB [8,10,70,71,72]. It was surprising that, about 15 weeks after the challenge, animals in the control group with large pathological damage in the lungs and lymph nodes and lesion scores of 10 at euthanasia showed no signs of TB and had similar life behaviors to those of the vaccinated animals. In some countries, there is a popular belief that goats are naturally resistant to TB; however, there is no scientific evidence that supports this hypothesis.

Our results show that vaccination protocols with BCG and boosting with proteins has the potential to reduce the dissemination of TB in animal populations by reducing the pathological damage in those vaccinated. This supports the hypothesis that vaccinations, incorporated into the current control programs, could be useful to accelerate the elimination of this disease in infected herds. Our working group is now prepared to go to the next step, large-scale field trials in commercial herds.

## 5. Conclusions

This study shows that BCG vaccine priming with CFP + Montanide^TM^ boost or chitosan-coated BCG priming with CFP + Montanide boost + PGLA reduces the pathological damage in vaccinated, compared to not vaccinated, goats. These findings can potentially be extended to other animal species.

## Figures and Tables

**Table 1:** Experimental groups to determine the efficacy of the BCG vaccine in different protocols of vaccination in a goat model. CFP: culture filtrate protein and PLGA: PolyLactic-co-Glycolic Acid.

Group Number	Priming Formulation	Boosting Formulation	Boosting Adjuvant
1	None	None	None
2	BCG	None	None
3	BCG	CFP	Montanide^TM^
4	Chitosan-coated BCG	CFP + Chitosan + PLGA	Montanide^TM^
5	Chitosan- coated BCG	CFP + Chitosan	Montanide^TM^

**Table 2:** Definition of lesion scores in the carcass inspections at slaughter of goats vaccinated against tuberculosis (TB) with different BCG vaccine protocols and challenged with a wild-type strain of *Mycobacterium bovis*.

Score	Score Definition
0	No visible lesions
1–1.9	Few lesions (≤20) in lung or lymph nodes
2–2.9	Few lesions (≤20) in lung and lymph nodes
3–3.9	Between 21 and 50 lesions in lung or lymph nodes
4–4.9	Between 21 and 50 lesions in lung and lymph nodes
5–5.9	Multiple lesions (between 51 and 100) localized in lung or lymph nodes
6–6.9	Multiple lesions (between 51 and 100) localized in lung and lymph nodes
7–7.9	Multiple lesions (≥101) disseminated in lung or lymph nodes
≥8	Multiple lesions (≥101) disseminated in lung and lymph nodes.

**Table 3:** Average, standard deviation, and 95% confidence intervals for the means of IFN-γ release for all experimental groups of goats vaccinated and challenged with a wild-type *Mycobacterium bovis* strain at each sampling week.

Sampling Week	Experimental Group	Mean * IFN-γ Release (OD 450 nm)	Standard Deviation	95% CI	*p*-Value
1	1	0.1866 ^a^	0.2419	−0.0672; 0.4405	0.713
Prime BCG vaccination	2	0.0914 ^a^	0.0164	0.0762; 0.1066	
	3	0.0970 ^a^	0.0366	0.0361; 0.1308	
	4	0.1661 ^a^	0.2277	−0.0440; 0.3767	
	5	0.1101 ^a^	0.0751	0.0313; 0.1890	
3	1	0.01453 ^a^	0.0350	0.1085; 0.1821	0.25
Groups 3–5 boosting	2	0.2711 ^a^	0.0350	−0.0340; 0.5763	
	3	0.3282 ^a^	0.1801	0.1616; 0.4949	
	4	0.2578 ^a^	0.1251	0.1421; 0.3736	
	5	0.1245 ^a^	0.0331	0.0897; 0.1592	
6	1	0.1235 ^a^	0.0367	0.0849; 0.1620	0.357
All groups challenging	2	0.1377 ^a^	0.0772	0.0662; 0.2091	
	3	0.2185 ^a^	0.1406	0.0884; 0.3486	
	4	0.2197 ^a^	0.2122	0.0233; 0.460	
	5	0.1158 ^a^	0.0275	0.0869; 0.1447	
8	1	0.2021^b^	0.0180	0.1831; 0.2211	0.0001
	2	0.0947 ^a^	0.0106	0.0849; 0.1044	
	3	0.1114 ^a^	0.0352	0.0788; 0.1440	
	4	0.0925 ^a^	0.0366	0.0586; 0.1264	
	5	0.1495 ^a,b^	0.0663	0.0798; 0.2191	
11	1	0.1755 ^a^	0.0436	0.1296; 0.2213	0.05
	2	0.3252 ^a,b^	0.0759	0.2550; 0.3954	
	3	0.5140 ^b^	0.1366	0.3877; 0.6402	
	4	0.3640 ^a,b^	0.2260	0.1750; 0.5529	
	5	0.4252 ^a,b^	0.3734	−0.0385; 0.8889	
13	1	0.0988 ^a^	0.0204	0.0773; 0.1203	0.049
	2	0.2364 ^a^	0.1450	0.1022; 0.3705	
	3	0.2405 ^a^	0.0820	0.1647; 0.3164	
	4	0.1848 ^a^	0.1010	0.0914; 0.2783	
	5	0.1475 ^a^	0.0401	0.1053; 0.1896	
16	1	0.2556 ^a^	0.1852	0.0612; 0.4501	0.002
	2	0.4712 ^a,b^	0.2387	0.2504; 0.6921	
	3	0.3667 ^a,b^	0.2463	0.1389; 0.5945	
	4	0.7475 ^b^	0.4321	0.3478; 1.1472	
	5	0.1013 ^a^	0.04951	0.0493; 0.1532	
18	1	0.0933 ^a^	0.0205	0.0717; 0.1149	0.002
	2	1.1730 ^b^	0.8178	0.4166; 1.9293	
	3	0.6585 ^a,b^	0.4943	0.2014; 1.1157	
	4	0.4650 ^a,b^	0.3746	0.1184; 0.8115	
	5	0.1053 ^a^	0.0307	0.0730; 0.1375	
20	1	0.7015 ^a,b^	0.2699	0.4181; 0.9848	0.050
	2	1.022 ^a,b^	0.6780	0.3949; 1.6490	
	3	0.7787 ^a,b^	0.5017	0.3146; 1.2427	
	4	1.4491 ^b,c^	0.8747	0.6401; 2.2581	
	5	0.4566 ^a,b^	0.3581	0.0807; 0.8325	
22	1	0.2473 ^a^	0.1335	0.1072; 0.3874	0.001
	2	0.7285 ^a,b^	0.5109	0.2560; 1.2011	
	3	0.4198 ^a,b^	0.3974	0.0522; 0.7874	
	4	1.2011 ^b^	0.7193	0.5357; 1.8665	
	5	0.1071 ^a^	0.0343	0.0711; 0.1432	
24	1	0.4270 ^a^	0.2106	0.2059; 0.6480	0.137
	2	0.3648 ^a^	0.2789	0.1068; 0.6228	
	3	0.1421 ^a^	0.0583	0.0881; 0.1960	
	4	0.4712 ^a^	0.4152	0.0872; 0.8553	
	5	0.2450 ^a^	0.1186	0.1204; 0.3695	
28	1	0.3918 ^a^	0.3512	0.0232; 0.7604	0.158
	2	0.1785 ^a^	0.0802	0.1043; 0.2528	
	3	0.1441 ^a^	0.0780	0.0719; 0.2163	
	4	0.2178 ^a^	0.1717	0.0590; 0.3676	
	5	0.2430 ^a^	0.0725	0.1668; 0.3191	
30	1	0.0688 ^a^	0.0138	0.0542; 0.0833	0.180
Euthanasia	2	0.1361 ^a^	0.0556	0.0847; 0.1875	
	3	0.1174 ^a^	0.0529	0.0684; 0.1663	
	4	0.1205 ^a^	0.0498	0.0744; 0.1667	
	5	0.0665 ^a^	0.0079	0.0581; 0.0748	

* Means with similar literals are not statistically different in an honest significant difference (HSD) Tukey’s test (*p* > 0.05).

**Table 4:** Number and percentage of animals with visible lesions, average, standard deviation, and 95% confidence intervals for lesion scores after vaccination against TB and challenged with a wild-type strain in a goat model.

Experimental Group	Animals with Lesion */Animals Challenged (%)	Average Lesion Score **	95% CI
1	6/6 (100)	6.33 ^b^ ± 3.2	2.97; 9.69
2	6/7 (86)	3.00 ^ab^ ± 2.2	0.98; 5.01
3	2/7 (28)	1.07 ^a^ ± 2.2	−1.00; 3.14
4	2/7 (28)	1.43 ^a^ ± 3.2	−1.51; 4.35
5	4/6 (67)	1.75 ^a^ ± 1.5	0.20; 3.29

* Lesions in lungs and/or lymph nodes (retropharyngeal, tracheobronchial, mediastinal, and mesenteric). ** Average lesion scores with similar literals are not statistically different in an HSD Tukey’s test (*p* >0.05).
